# Stable and tunable expression of human peripheral myelin protein 22 in Rat Schwann cells

**DOI:** 10.1016/j.jbc.2026.113270

**Published:** 2026-06-19

**Authors:** Mason C. Wilkinson, Katherine M. Stefanski, Pramod S. Gowda, Bruce D. Carter, Charles R. Sanders

**Affiliations:** 1 Department of Biochemistry, Vanderbilt University School of Medicine, Nashville, Tennessee, USA; 2 Center for Structural Biology and Institute for Chemical Biology, Vanderbilt University School of Medicine, Nashville, Tennessee, USA; 3 Vanderbilt Brain Institute, Vanderbilt University School of Medicine, Nashville, Tennessee, USA

**Keywords:** PMP22, myelin, Charcot-Marie-Tooth disease, peripheral myelin protein 22, trafficking, mistrafficking, HNPP, CMT1A, folding, misfolding, membrane protein, overexpression

## Abstract

Duplications and deletions of the gene encoding peripheral myelin protein 22 (PMP22) cause the most common forms of Charcot-Marie-Tooth (CMT) disease: type 1A (CMT1A) and hereditary neuropathy with liability to pressure palsies (HNPP), respectively. The resulting over- or under-expression of PMP22 in Schwann cells (SCs) causes myelin defects in the peripheral nervous system (PNS). Although the roles of PMP22 in myelin structure and maintenance have been studied extensively, the mechanisms by which perturbed PMP22 expression levels cause dysmyelination are not yet fully understood. We therefore developed a clonal rat Schwann cell (RSC) line that can express myc-tagged human PMP22 from a doxycycline-inducible genomic locus. MycPMP22 expression levels in this cell line can be tuned by adjusting the doxycycline (DOX) concentration in culture media, achieving maximal expression between 800 to 1600 ng/ml. The percentage of the total protein that reached the plasma membrane was found to inversely correlate with expression level. At high expression levels, intracellularly retained mycPMP22 localizes to puncta that can be ameliorated by serum starvation. These observations support the hypothesis that overexpression of PMP22 in CMT1A causes increased misfolding accompanied by formation of intracellular inclusions. Moreover, this work enables the precise control of mycPMP22 levels for studying CMT-related phenomena in SCs.

Charcot-Marie-Tooth disease is the largest group of heritable neuropathies affecting the peripheral nervous system (PNS), afflicting approximately one in 2500 people in western populations (1, 2, 3, 4). 80% of all CMT cases represent the demyelinating form of CMT, resulting in degeneration of PNS axons and reduced peripheral nerve function (5, 6). While the etiology, pathology, and severity of different forms of CMT can vary, debilitating symptoms such as reduced nerve conduction velocity, muscle weakness and atrophy, and *pes cavus* (highly arched feet) usually appear in early adolescence and worsen progressively (5). No treatments currently exist for any form of CMT and much remains unknown regarding its pathological mechanisms (2, 7, 8).

Roughly two-thirds of CMT cases are caused by pathogenic mutations affecting the gene encoding PMP22, a tetraspan transmembrane glycoprotein that is highly expressed by myelinating SCs (4, 9, 10). SCs are the glia responsible for forming the myelin sheath of the PNS (3, 5, 11, 12, 13). PMP22 is believed to serve critical roles in myelin formation, maintenance, and stability, as well as in intracellular cholesterol transport, growth arrest, and cell cycle regulation in SCs (11, 12, 13, 14, 15, 16, 17, 18). Duplication or deletion of the PMP22 gene results in CMT1A and HNPP, respectively, while point mutations can cause CMT type 1E (CMT1E) (5, 11, 19). Of these subtypes, CMT1A is the most common (ca. 55% of all CMT cases) and is associated with substantial demyelination of peripheral axons and moderate progressive neuropathy (4, 5, 7, 9, 10). Despite its frequency, the etiology and pathophysiology of CMT1A remain incompletely understood.

WT PMP22 folds inefficiently in model cell lines like HEK293, where the misfolded protein either accumulates inside the cells as inclusions or is degraded by proteasomal and autophagy/lysosomal pathways (1, 16, 18, 20, 20, 20, 21, 22, 23, 24, 25). The fraction of WT PMP22 that reaches the cell surface has been reported to be in the range of 20% to 50% (20, 23, 26, 27, 28, 29). In transiently transfected HEK 293 and MDCK cells, higher PMP22 expression leads to more severe misfolding and mistrafficking of WT PMP22, with disproportionately higher intracellular accumulation compared to surface expression (26).

Based in part on the above observations, excessive PMP22 misfolding and mistrafficking during overexpression have been implicated in CMT1A pathogenesis (11, 20, 26, 30, 31, 32). However, the molecular details of how PMP22 drives disease remain poorly understood, in part, due to the limitations of the available experimental model systems. Nevertheless, the relevance of the model cell line observations summarized in the above paragraph has been supported by observation of increased levels of intracellular PMP22 in a variety of CMT1A-relevant cell lines, including those from CMT1A patients (20, 22, 23, 24, 32, 33). For a comprehensive review of PMP22’s roles in myelin and CMT, see (34).

There remains a need for more quantitative studies of PMP22 folding, misfolding, and trafficking in SCs. However, isolated SCs in culture do not usually express PMP22, and the transient transfection of SCs does not allow for precise control of expression levels. We were therefore motivated to create a cell line that overcomes these limitations. Here, we describe the generation by lentiviral transduction of a clonal rat SC (RSC) line that stably expresses myc-tagged human PMP22 (mycPMP22) upon the addition of doxycycline (DOX) to the cell culture medium. MycPMP22 expression levels within these cells can be tuned to low, intermediate, or high levels by adjusting the doxycycline concentration in the cell culture medium. We then tested the dependence of mycPMP22 surface trafficking *versus* intracellular entrapment as a function of total expression levels. We found that mycPMP22 trafficking efficiency inversely correlates with its expression level in the clonal RSCs, that intracellular mycPMP22 puncta accumulate at high expression levels, and that these puncta can be ameliorated *via* serum starvation.

## Results

### Generation of a clonal RSC line for inducible, tunable expression of myc-tagged human PMP22

**Figure 1:**
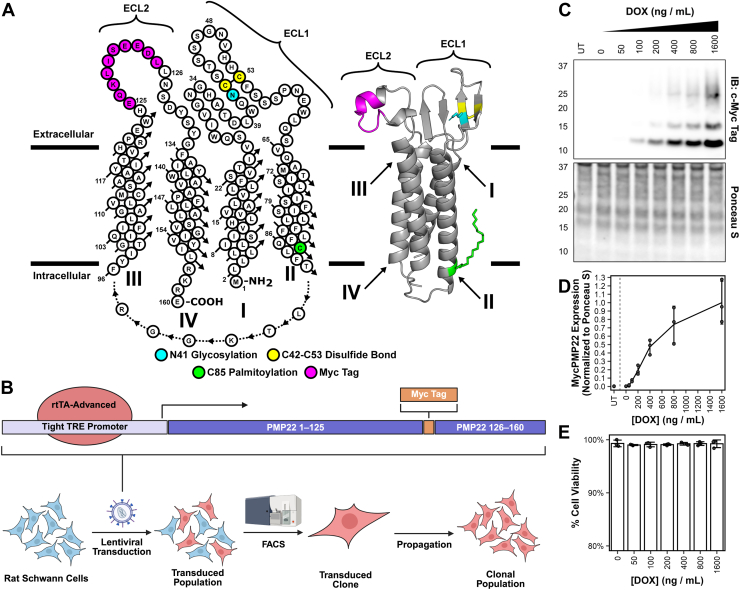
**Stable, inducible expression of mycPMP22 in Rat Schwann cells is tunable and non-toxic**. *A*, topology diagram (*left*) and AlphaFold three model (*right*) of human PMP22 with a 10-residue myc tag (*pink*) inserted into its second extracellular loop. PMP22’s four transmembrane helices are labeled I–IV. Known or putative sites of post-translational modifications are colored as indicated. The 3D model of C85-palmitoyl mycPMP22 was generated using the AlphaFold three web server (39, 40). *B,* workflow used in generating clonal RSCs stably expressing the myc-tagged human PMP22 gene flanked by a tetracycline-inducible promoter. This panel was created in BioRender. Wilkinson, M. (2026) https://BioRender.com/czhij0h. *C,* Western blot of clonal RSCs treated with the indicated concentrations of doxycycline for 48 h. The three anti-myc immunoreactive bands represent non-glycosylated PMP22 (observed ∼ 14 kDa; actual ∼ 18 kDa), the immature, high-mannose glycoform (observed ∼ 16 kDa; actual ∼ 22 kDa), and mature, complex-glycosylated forms of mycPMP22 (observed ∼ 20–37 kDa). *D,* quantification of anti-myc tag immunoblot signals (all bands) from *panelB*. Myc tag immunoblot signals are normalized to the average value of the 1600 ng/ml DOX treatment group. *E,* cell viability at the indicated DOX concentrations. Data points in *panelsC* and *D* indicate values are from individual biological replicates (n = 3). Line traces and error bars indicate the means ± SDs. DOX, doxycycline; FACS, fluorescence-activated cell sorting; MycPMP22, myc-tagged human PMP22; rtTA, reverse tetracycline-controlled transactivator protein; SD, standard deviation; UT, untransduced RSCs.

To establish an RSC line capable of stable and inducible PMP22 expression, RSCs were transduced with lentivirus containing cDNA that encodes myc-tagged human PMP22 under the control of a tight TRE promoter, a constitutively expressed puromycin resistance gene, and the tetracycline transactivator protein, rt-TA Advanced (AKA rtTA2S-M2) (Figs. 1, *A* and *B* and S1) (35). The 10-residue myc tag was inserted between residues 125 and 126 in the second extracellular loop of PMP22, as this internal tag was previously demonstrated to be non-perturbative of PMP22 folding and trafficking (Fig. 1*A*) (36, 37). This resulted in a heterogeneously transduced RSC population with varying levels of human mycPMP22 expression and DOX-inducibility. It should be noted that under non-myelinating conditions in culture, the level of endogenous rat PMP22 expression is negligible (23, 38).

The heterogeneous population was first induced with DOX then surface-stained with an Alexa Fluor 488 (AF488)-conjugated anti-myc tag antibody to label the plasma membranes of antibody-impermeant positively transduced RSCs expressing mycPMP22. Individual AF488-positive RSCs were next isolated *via* fluorescence-activated cell sorting (FACS) and propagated into clonal populations for approximately one month before further analysis (Fig. 1*B*). Individual clones were assessed for DOX-inducible mycPMP22 expression by anti-myc tag Western blot or immunofluorescence microscopy. One exemplar clone was selected for its high level of mycPMP22 expression at full induction and minimal expression in the absence of DOX (Figs. 1, *C* and *D* and S2). Indeed, induction with 1600 ng/ml DOX for 48 h resulted in an approximately 400-fold increase in mycPMP22 expression over uninduced cells (Fig. 1*D*). Next, we quantified clonal RSC viability as a function of PMP22 level and did not observe overexpression-induced toxicity after 48 h of treatment with any of the tested DOX concentrations (Fig. 1*E*). As both mature and immature mycPMP22 glycoforms can be seen in the Western blot from Figure 1*C* (see caption for explanation), we next sought to address aspects of mycPMP22 trafficking and mistrafficking that have been reported in other cell lines.

### Overexpression reduces the fraction of mycPMP22 protein at the plasma membrane

**Figure 2:**
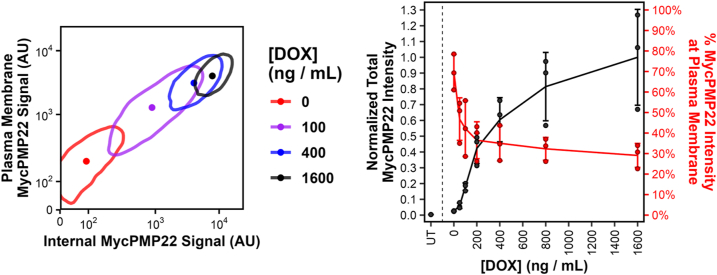
**The fraction of mycPMP22 at the plasma membrane decreases as total expression increases**. *Left,* 2D-density plot of a *representative* two-color flow cytometry trafficking assay experiment depicting signal from mycPMP22 within the cell vs. at the plasma membrane. Groups are colored by DOX treatment concentration. Points depict the median value within a population of cells (13,709–19,035 cells per treatment condition). Boundaries encompass 67.5% of all cells within a population (2D Gaussian 1-sigma confidence interval centered around the median value). *Right,* Total mycPMP22 signal intensity (*black*) and the fraction of the total present at the plasma membrane (*red*) was assessed by a two-color flow cytometry assay. All intensity values are normalized to the average mycPMP22 signal value for the 1600 ng/ml DOX treatment group. Data points indicate the median values of all cells from individual biological replicates (n = 3). Line traces and error bars indicate the means ± SDs values between replicates.

We first examined mycPMP22 plasma membrane trafficking. It has previously been reported that the degree of intracellular retention of mycPMP22 point mutants correlates with disease severity, and that WT PMP22 is increasingly and disproportionately retained as a function of its expression level (11, 20, 26). As such, we sought to determine whether the inducible RSCs recapitulate this result using a two-color flow cytometry-based assay to quantify and differentiate mycPMP22 levels at the plasma membrane *versus* within the cell (28). MycPMP22 at the plasma membrane is differentiated from internal mycPMP22 by staining live RSCs with a phycoerythrin (PE)-conjugated anti-myc tag antibody, then fixing and permeabilizing the cells. This process blocks myc tag antigens at the plasma membrane with the PE label. Next the cells are reprobed with an Alexa Fluor 647 (AF647)-conjugated anti-myc tag antibody, which can only label unblocked (intracellular) myc tag epitopes. Subsequent fluorophore brightness correction allows us to calculate the total mycPMP22 expression and the fraction of mycPMP22 molecules that are present at the plasma membrane (see Supporting Information for extended methods and analysis). As expected, both intracellular and plasma membrane mycPMP22 signals increase as a function of DOX concentration (Fig. 2, *Left*; Figs. S3 and S4). Note that while the *absolute* cell-to-cell variance in mycPMP22 fluorescence scales with the DOX concentration, the *relative* variance decreases as more cells begin to converge on a high level of expression at maximal induction (see also both Table S2 and the Excel data in the Supporting Information).

Most importantly, it was observed that while the mean fraction of plasma membrane to total cellular mycPMP22 from leaky expression in uninduced cells was 70 ± 9%, this value fell to 46 ± 10% upon induction with 50 ng/ml DOX and dropped even further to 29 ± 6% upon maximal induction with 1600 ng/ml DOX (Fig. 2, *Right*). This latter value is near the previously reported 27 ± 1% value observed in transiently transfected HEK 293 cells overexpressing mycPMP22 (26).

### MycPMP22 puncta formation correlates with the expression level

**Figure 3:**
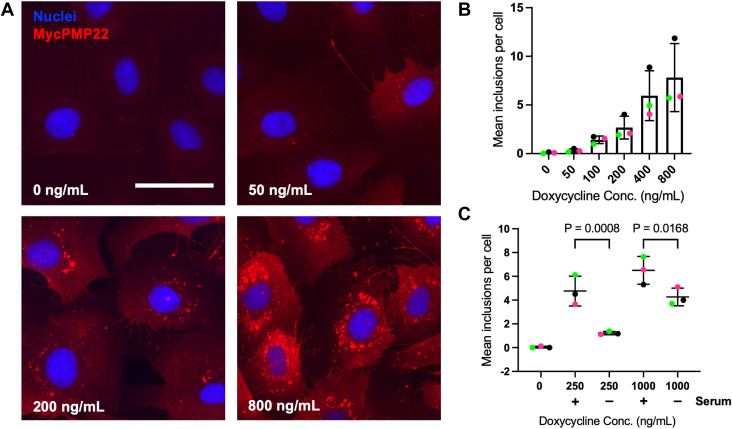
**PMP22 inclusions increase with doxycycline induction**. *A,* representative images of mycPMP22-inducible RSCs treated with increasing amount of DOX and cultured for 48 h mycPMP22 in immunolabeled *via* the myc epitope. Images are cropped from 60X fields of view. Scale bar = 50 μm, scale is identical for all panels. *B,* quantification of puncta visible in *A*. 12 to 18 images per treatment were acquired for each of three biological replicates. *C,* quantification of puncta in cells induced with indicated levels of DOX ± serum starvation. All cells were first induced for 24 h in complete medium, then “Serum (−)” cells were then placed in serum-free medium for another 24 h with the indicated amount of DOX. All cells were labeled and imaged as in *B* after 48 h of total induction. Bars in *B* and *C* indicate means ± SDs, while points are from each of three individual biological replicates and grouped by color. A one-way ANOVA was conducted (F test, *p* < 0.0001) and *p*-values for pairwise comparisons were calculated from Bonferroni’s multiple comparison *post hoc* tests.

We next examined the formation of intracellular mycPMP22 puncta using immunofluorescence microscopy. Such puncta represent another phenomenon previously reported to be associated with CMT1A and CMT1E (20, 22, 33, 41, 42, 43). The inducible RSCs provided an opportunity to observe formation of mycPMP22 puncta under controlled expression. We therefore induced mycPMP22 expression for 48 h with DOX ranging from 0 to 800 ng/ml and immunolabeled mycPMP22 for imaging and quantified puncta from the resulting micrographs. We observed few to no puncta at low levels of mycPMP22 expression and abundant puncta at higher levels of mycPMP22 expression (Fig. 3*A*). Quantification revealed a steady increase in puncta in response to DOX levels (Fig. 3*B*). These data, combined with the trafficking dose-response data, suggest that mistrafficked PMP22 may largely accumulate in these intracellular puncta.

We further observed that under serum starvation conditions, mycPMP22 puncta still accumulate, especially at high induction levels, but are significantly decreased in number relative to the non-starved condition (Fig. 3*C*). This may be the result of less mycPMP22 being synthesized under serum starvation or of it being efficiently cleared *via* autophagy. Again, these experiments demonstrate that this cell line recapitulates a previously described phenomenon and allowed us to make dose-response observations.

## Discussion

PMP22 mistrafficking following overexpression is believed to be the primary driver of CMT1A pathology but remains incompletely understood. Current models such as explanted rodent primary SCs, SC-neuron co-cultures, dorsal root ganglion (DRG) explant cultures, or induced pluripotent stem cell (iPSC)-derived human SCs have successfully been used to study aspects of PMP22 trafficking and myelination, though these strategies come with substantial burdens in terms of time and reagents (32, 44, 45, 46, 47). We have therefore developed a tunable, inducible RSC model to facilitate the study of PMP22 expression in isolation through an easily maintainable, approachable, and physiologically relevant cell line.

### Inducible mycPMP22 expression levels are tunable, and overexpression causes its intracellular accumulation

We have demonstrated that mycPMP22 expression can be tightly controlled by varying the DOX concentration of the cell culture media. Furthermore, our results demonstrate that DOX-induced PMP22 overexpression in these RSCs reduces the fraction of mycPMP22 at the plasma membrane and increases its intracellular sequestration in a dose-dependent manner. Most notably our results indicate that as total mycPMP22 expression levels increase, the population of mycPMP22 that is trapped intracellularly increases disproportionately to corresponding increases in PMP22 levels at the cell surface. Consequently, the mycPMP22 surface trafficking efficiency (surface/total X 100) is dramatically reduced from roughly 70% under very low expression conditions to roughly 30% at higher levels of expression. Indeed, a decrease to <40% efficient expression is seen to be complete even at a 200 ng/ml DOX concentration, where the total mycPMP22 expression level is roughly 50% that of the 1600 ng/ml DOX condition. Thus, mycPMP22 trafficking efficiency appears sensitive even to expression levels that are relatively modest.

Intracellularly trapped mycPMP22 is readily observed at all but the lowest expression levels in the form of puncta. Whether these puncta represent aggregated mycPMP22 within aggresomes or some other form of mycPMP22-rich condensate is not yet clear. Also not known is whether these inclusions contain other molecules in addition to mycPMP22. However, our observations are consistent with previous results in which intracellular mycPMP22 inclusions have been seen in PMP22-overexpressing mouse peripheral nerves, in CMT1A patient-derived fibroblasts, and in other relevant cells (20, 22, 23, 24, 32, 33). Previous results from our lab suggest that these puncta are formed by mistrafficked PMP22 and may drive CMT1A pathology (29).

We also observed that serum starvation significantly decreases the number of puncta formed by cells of the new line, possibly by downregulating expression to prevent initial accumulation or by inducing autophagy to improve the clearance of existing puncta, as has been previously demonstrated in primary RSCs (22). The puncta data, combined with the trafficking data, demonstrate that these cells recapitulate previously observed CMT-related phenomenon. Additionally, since the investigator has control over the amount of mycPMP22 synthesized in these cells, these phenomena can be examined as they relate directly to levels of mycPMP22 expression in a dose-response manner.

### Limitations of the clonal doxycycline-inducible RSC line

While our RSC model addresses some shortcomings of other cell culture models, it does have several potential limitations. First, the expression and trafficking of mycPMP22 in isolation likely do not reflect the regulatory environment within actively myelinating SCs. Second, the genomically integrated mycPMP22 gene is intron-less, has a non-native promoter system, and an unknown site of integration, thereby obviating the use of these cells in studying transcriptional regulation of PMP22. Third, these RSCs have been cultured and passaged without neuronal contact for at least one month. As such, some degree of dedifferentiation and/or phenotypic drift is expected. For instance, as shown in Figure 3*A*, the RSCs have lost the swirled, spindly morphology that is characteristic of primary myelinating SC cultures. While we have not investigated the biological similarity between our model RSCs and freshly isolated primary myelinating SCs, it is likely that the myelinating capacity of the model RSCs is reduced if not outright eliminated. Fourth, while the myc tag has previously been demonstrated to be non-perturbative of PMP22 folding and trafficking, PMP22 is believed to be a multi-functional protein, and it cannot be yet be ruled out that the presence of the 10-residue myc tag in its second extracellular loop could attenuate some of its functions (36, 37). Lastly, it is not clear which SC mycPMP22 expression level(s) observed in this study correspond to those for PMP22 being expressed in myelinating SCs under healthy, CMT1A overexpression, or HNPP underexpression physiological conditions (38, 48, 49, 50, 51). In all three cases measurement of PMP22 expression levels in PNS tissue is not straightforward for reasons reviewed in (34).

In summary, many details of how PMP22 over- or underexpression ultimately leads to CMT1A or HNPP disease symptoms remain unclear, but we suggest that our DOX-inducible RSCs provide an approachable system to study PMP22 at defined expression levels in a native-like model system. Here we demonstrate that increased total mycPMP22 expression is accompanied by reduced mycPMP22 cell surface trafficking efficiency and increased numbers of intracellular inclusions. This cell line may be particularly useful for future microscopic and proteomic studies investigating PMP22 interactors and intracellular localization. Such studies could shed light on poorly characterized aspects of PMP22’s folding and trafficking pathways, its turnover and degradation, and its role in cholesterol trafficking to the plasma membrane. Also, because cell-to-cell expression levels are much more constant in a stable cell line, this work may also provide a useful tool for CMT drug discovery efforts that involve a search for compounds that modulate PMP22 expression levels for therapeutic use in CMT1A, CMT1E, or HNPP patients.

## Experimental procedures

### Isolation and culture of rat Schwann cells

Primary SCs were isolated from P2-P3 Sprague-Dawley rats (*Rattus norvegicus domestica*) as described previously using methods from Chan *et al.* (2004) (52, 53). RSCs were maintained in collagen-coated (Sigma-Aldrich #C3867D) tissue culture dishes containing high-glucose Dulbecco’s Modification of Eagle’s Medium with L-glutamine and pyruvate (DMEM) (Gibco, 11,995,065) supplemented with 10% tetracycline-negative FBS (Corning, 35–075-CV) and 2 μM forskolin (Sigma-Aldrich, F3917) in a humidified incubator at 37 °C with 5% atmospheric CO_2_. RSCs were passaged at a ratio of 1:4–1:10 as needed (every 2–5 days). Clonal lentiviral RSC populations were only used for experiments up to 20 passages post-sorting (see below).

### Cloning and lentiviral particle production

A DNA fragment encoding an intron-less human PMP22 ORF (NM_153321) with an internal myc epitope tag (EQKLISEEDL) inserted between residues 125 and 126 was synthesized and subcloned into the pCW57.1 vector (Addgene, 41393) between NheI and AgeI restriction sites (Integrated DNA Technologies (IDT)). Lentiviral particles were produced by co-transfection of HEK 293T cells with pCW57.1-Myc-hPMP22 alongside the second-generation packaging plasmid psPAX2 (Addgene, 12260) and the VSV-G envelope plasmid pMD2.G (Addgene, 12259) using Lipofectamine 3000 Transfection Reagent (Invitrogen, L3000001) per the manufacturer’s protocol. Conditioned medium was collected after 48 to 72 h post-transfection, clarified by filtration, and used immediately for transduction.

### Lentiviral transduction

RSCs were seeded in 6-well dishes at 0.5 x 10^6^–1.5 x 10^6^ cells/well then transduced sequentially with 1 ml of filtered viral supernatant 24 and 48 h post-seeding. Following transduction, RSCs were maintained in complete medium supplemented with 5 μg/ml puromycin (Sigma-Aldrich, P7255) for 3 days to select for cells with the genomic integration prior to downstream experimentation or cryopreservation.

### Isolation of clonal transduced RSC populations expressing myc-tagged human PMP22

The bulk transduced RSC population was seeded into a 100 mm cell culture dish at a density of 3725 cells/cm^2^ and grown for 24 h. Conditioned medium was then replaced with fresh complete medium supplemented with 1 μg/ml DOX (Sigma-Aldrich, D5207) and the cells were incubated for an additional 48 h. Cells were resuspended using 0.25% trypsin-EDTA (Gibco, 25,200–056), washed with PBS, and resuspended in 100 μl of complete media containing 0.75 μg/ml Alexa Fluor 488 (AF488)-conjugated mouse anti-myc tag monoclonal IgG (Cell Signaling Technology, 2279) for 15 min at 37 °C with 5% atmospheric CO_2_. Cells were washed twice with PBS, resuspended in 500 μl of complete media containing 1 μg/ml propidium iodide (PI) (Sigma-Aldrich, P4864), and transferred to sterile flow cytometry tubes. Individual AF488-positive cells were isolated by FACS into 96-well plates containing 100 μl complete media using a BD FACSAria III equipped with a 100 μm nozzle. Clones were propagated to larger cultures for roughly one month before experimentation or cryopreservation in complete media containing 10% DMSO (Sigma-Aldrich, D8418). Clonal populations were evaluated for inducible PMP22 expression by Western blot or immunofluorescence microscopy (see relevant sections). Following validation, clonal populations were maintained in complete media without puromycin for up to 20 passages.

### Western blotting, cell viability assay, and two-color flow cytometry mycPMP22 trafficking assay

Details for these methods appear in the Supporting Information.

### Puncta quantification

RSCs were seeded into a 96-well plate (Greiner, 655,090) and grown for 24 h mycPMP22 expression was then induced with indicated DOX concentrations for 48 h, after which cells were fixed with 4% paraformaldehyde (ChemCruz, SC-281692) and permeabilized with 0.3% Triton X-100 (Sigma-Aldrich, X100). MycPMP22 was immunolabeled *via* the myc tag using a mouse anti-myc tag primary IgG (Cell Signaling Technology, 2276, validated for specificity by manufacturer) at a 1:1000 dilution (80 ng/ml), followed by an AF647-conjugated goat anti-mouse IgG F(ab')2 fragment (Cell Signaling Technology, 4410, validated for specificity by manufacturer) at a 1:2000 dilution (1 μg/ml). Nuclei were labeled with DAPI (BD Pharmingen, 564907). Plates were imaged on an ImageXpress Micro Confocal High Content Screening System (Molecular Devices) with a Nikon, 60x 0.95 numerical aperture Plan Apo Lambda Objective, an Andor Zyla 4.2 MP 83% QE sCMOS camera, and an 89-North LDI five channel laser light source. Six to nine fields were imaged per well in an unbiased manner. The plate was imaged using a spinning disc confocal module with a 60 μm pinhole size. All images were collected using identical laser powers and acquisition times. mycPMP22 puncta were quantified in MetaXpress (Molecular Devices). Briefly, images were segmented using the custom module editor using the granularity objects module. The channel corresponding to labeled PMP22 was used to identify puncta with using an approximate minimum size of 1 μm and approximate maximum size of 10 μm. Intensity thresholds were selected using control images and applied to every image.

### Data Plotting and Statistical Analysis

Data were analyzed and plotted using either GraphPad Prism 10 or R (version 4.5.0) with the tidyverse (version 2.0.0) and ggplot2 (version 3.5.2) packages (54, 55, 56). For one-way ANOVA, normality was confirmed by Shapiro-Wilk test in GraphPad Prism 10.

## Data availability

Supporting material containing expanded figures and uncut blots are provided. All strains and plasmids are available upon request.

## Supporting information

This article contains supporting information (28, 57, 58).

## Conflict of interest

The authors declare no conflicts of interest regarding the contents of this article.
