# Supplementary material for: Stable and tunable expression of human peripheral myelin protein 22 in Rat Schwann cells
Source: J Biol Chem. 2026 Jun 19;302(8):113270. doi: 10.1016/j.jbc.2026.113270 (PMC13382790; doi:10.1016/j.jbc.2026.113270)
Supplement: Supporting Information — 1 [file mmc1.pdf]

Supporting Information

Stable and Tunable Expression of Human Peripheral Myelin Protein 22 in Rat Schwann Cells

Mason C. Wilkinson[1,2,*], Katherine M. Stefanski[1,2,*], Pramod S. Gowda[1,3*],
Bruce D. Carter[1,3], Charles R Sanders[1,2]

[1]Department of Biochemistry, Vanderbilt University School of Medicine, Nashville, Tennessee, USA
[2]Center for Structural Biology and Institute for Chemical Biology, Vanderbilt University School of Medicine, Nashville, Tennessee, USA.
[3]Vanderbilt Brain Institute, Vanderbilt University School of Medicine, Nashville, Tennessee, USA.

* These authors contributed equally to this work
Corresponding author: Charles R. Sanders (chuck.sanders@Vanderbilt.Edu)

The Supporting Information contains:

Supporting Methods

Raw data underpinning main paper figures (as separate file):

Data from all figures.xlsx

Table S1: List of Strains

Table S2: Absolute and relative cell-to-cell variance in mycPMP22 expression values depicted in Figure 2 (*Left Panel*) and in Figure S3

Figure S1. Annotated lentiviral construct pCW57.1 (mycPMP22)

Figure S2. Uncropped membranes and additional GAPDH loading control immunoblot from Figure 1*B*

Figure S3. Expanded version of Figure 2 (*Left Panel*) highlighting additional DOX-induced RSC populations

Figure S4. Full population distributions of raw MycPMP22 immunofluorescence depicted in Figure 2 (*Left Panel*) and Figure S3

Supporting Methods

Western blot. RSCs were seeded into 60mm cell culture dishes at a density of 3725 cells/cm^2 and grown in a humidified incubator at 37ºC with 5% atmospheric CO_2 for 16-24 h. For lentiviral RSCs, the media was next replaced with fresh media supplemented with 0-1600 ng/mL DOX, then the cells were incubated at 37ºC with 5% atmospheric CO_2 for an additional 48 h. Media from the WT parent population RSCs was replaced similarly, but without DOX. Cells were harvested by scraping in ice-cold PBS and lysed in RIPA buffer (20 mM Tris-HCl, 136 mM NaCl, 1 mM EDTA, 1% IGEPAL CA-630, 0.5% Sodium Deoxycholate, 0.1% SDS, pH 7.4) supplemented with 1x mammalian protease inhibitor cocktail (Sigma-Aldrich, P8340), 1x Halt™ Phosphatase Inhibitor Single-Use Cocktail (Thermo Scientific, 78248), and 100 U/mL Benzonase® Nuclease (Millipore, E1014). Lysate concentrations were determined by BCA assay (Thermo Scientific, 23225). Reducing SDS-PAGE samples were prepared using NuPAGE LDS Sample Buffer (Invitrogen, NP0007) and 50 mM DTT (Sigma-Aldrich, D9779). 10 µg of total lysate protein per sample was loaded into 17-well 4-12% gradient polyacrylamide gels (Invitrogen, NP0329) and electrophoresed in ice-cold 1x NuPAGE MES SDS Running Buffer (Invitrogen, NP0002) at 100V for 2 h. Proteins were then transferred to 0.22 µm nitrocellulose membranes by semi-dry transfer using the Transblot Turbo Transfer System (Bio-Rad, 1704150) according to the manufacturer's instructions. Total protein levels were assessed by Ponceau S staining (Sigma-Aldrich, P3504). Membranes were probed with 80 ng/mL primary mouse anti-myc tag monoclonal IgG (Cell Signaling Technology, 2276) then detected with goat anti-mouse IgG HRP-conjugated secondary

antibody (Cell Signaling Technology, 7076) at a 1:3000 dilution. Chemiluminescent detection was facilitated using Clarity™ Western ECL Substrate (Bio-Rad, 1705060). Blot imaging was performed using an Amersham Imager 600 (GE Healthcare Life Sciences). Blot signals were quantified in FIJI (version 2.16.0) using the Band / Peak Quantification plugin (54, 55). All immunoblot signals were normalized to the corresponding Ponceau S intensity for each lane.

Cell viability assay. RSCs were plated, treated, trypsinized, and resuspended as in the Western blotting protocol. Cells were washed once with PBS to remove serum proteins and then stained with LIVE/DEAD™ Fixable Violet Dead Cell Stain (Invitrogen, L34955) for 30 minutes at room temperature before analysis with an BD LSRFortessa 5-laser analytical cytometer and analyzed in FlowJo (BD, LLC.). Paraformaldehyde-fixed RSCs were stained and used as a positive control for cell death. Viability was calculated as the percentage of single cell events that were negative for the LIVE/DEAD™ stain.

Two-color flow cytometry trafficking assay. The mycPMP22 trafficking assay was performed as described previously (28). Briefly, 1×10^5–1×10^6 RSCs were immunostained with a phycoerythrin (PE)-conjugated mouse anti-myc tag monoclonal IgG (Cell Signaling Technology, 3739). Cells were next fixed and permeabilized using the FIX & PERM™ Cell Permeabilization Kit (Invitrogen, GAS003) according to the manufacturer’s instructions. Intracellular mycPMP22 was next immunostained using an Alexa Fluor 647 (AF647)-conjugated mouse anti-myc tag monoclonal IgG (Cell Signaling Technology, 2233). Cells were analyzed using a BD LSRFortessa 5-laser

analytical cytometer and analyzed in FlowJo version 10. Untransduced RSCs were stained and used for background subtraction. Relative brightness correction between the two fluorophores was determined by comparing single color controls prepared from cells treated with 1600 ng/mL DOX. Total mycPMP22 signal is calculated as the sum of brightness-corrected PE and AF647 intensities (**Total = *PE + AF647***). Plasma membrane trafficking percentage is calculated PE intensity divided by the summed total intensity (***% Plasma Membrane Signal = PE / (PE + AF647)***).

Table S1: List of Strains

STRAIN	RELEVANT GENOTYPE	SOURCE
RSC Tet-ON Myc-hPMP22	*Tight-TRE_Myc-hPMP22 / hPGK_PuroR-T2A-rtTA-Advanced*	This work

Table S2. Absolute and relative cell-to-cell variance from median mycPMP22 expression values depicted in Figure 2 (*Left Panel*) and Figure S3. Absolute variation (median absolute deviation (MAD)) increases with DOX concentration, while relative variation (Robust Coefficient of Variation (CV = MAD / Median)) decreases.

[DOX] (ng/mL)	Plasma Membrane MycPMP22 Signal			Internal AF647-MycPMP22 Signal		
	Median (AU)	MAD (AU)	CV	Median (AU)	MAD (AU)	CV
**0**	209.4	171.3	0.82	91.6	91.1	0.99
**50**	478.3	455.5	0.95	392.0	344.2	0.88
**100**	1274.3	1082.4	0.85	890.5	940.7	1.06
**200**	2343.2	1554.4	0.66	3040.7	2074.5	0.68
**400**	3061.9	1761.0	0.58	3959.4	2675.4	0.68
**800**	3639.7	1811.2	0.50	6269.6	3101.8	0.49
**1600**	3959.5	1862.7	0.47	7783.6	3567.5	0.46

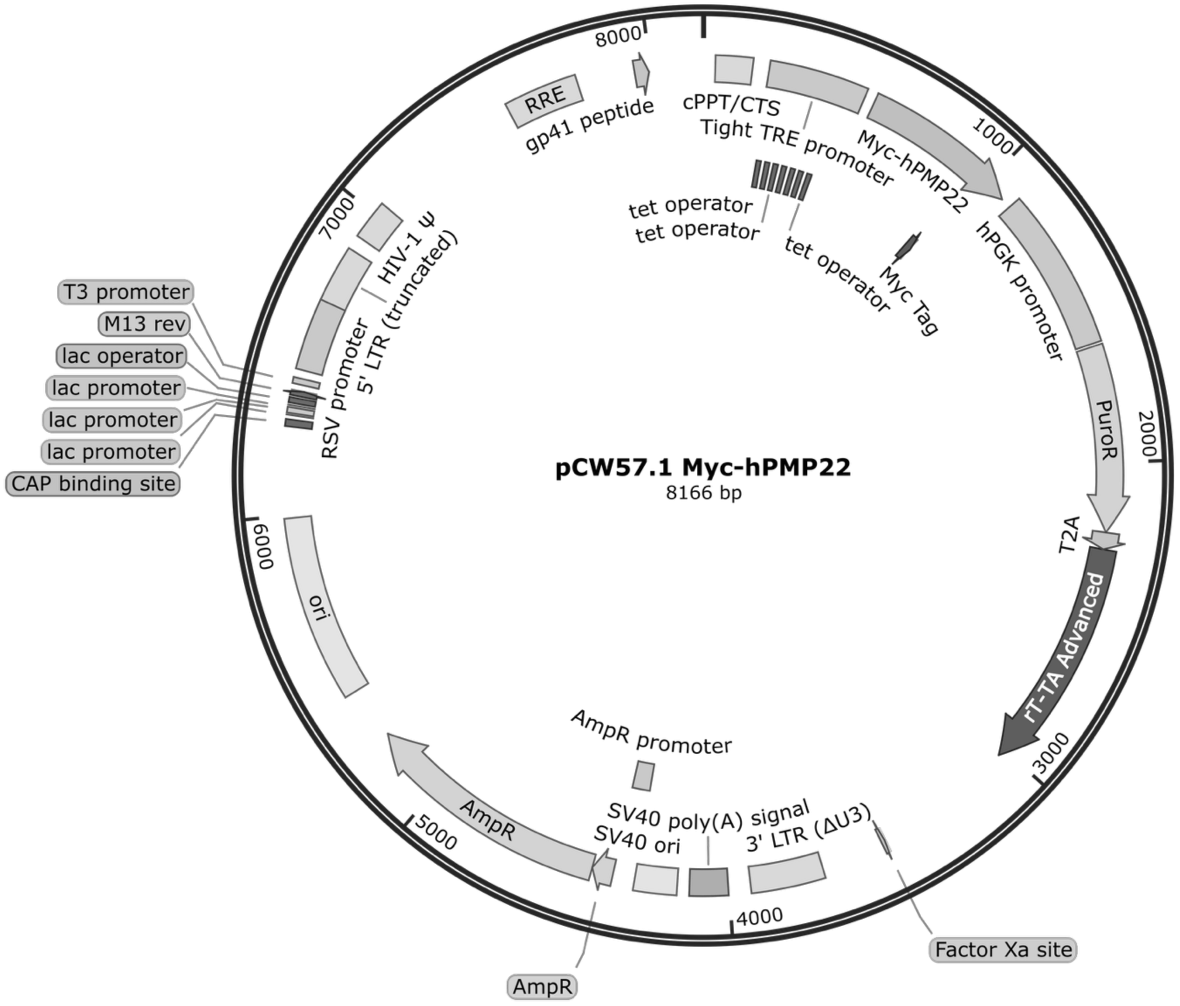

Figure S1. Annotated lentiviral construct pCW57.1 (mycPMP22). An intron-less human PMP22 ORF (NM_153321) with an internal myc tag (EQKLISEEDL) between residues 125 and 126 was first cloned into the pCW57.1 vector (Addgene 41393) between NheI and AgeI restriction sites. Vector map was prepared using the SnapGene software (version 8.1; www.snapgene.com).

Ponceau S

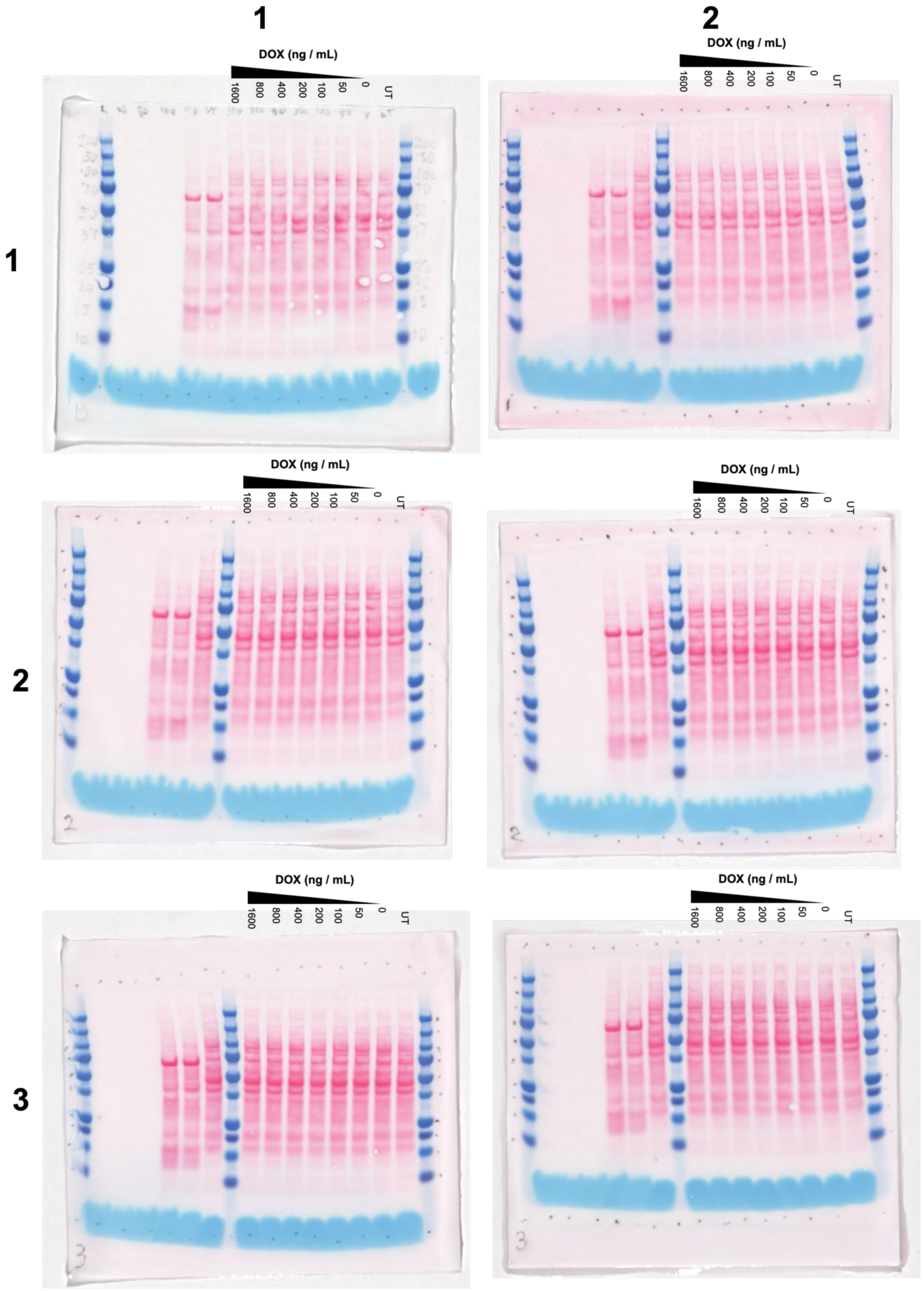

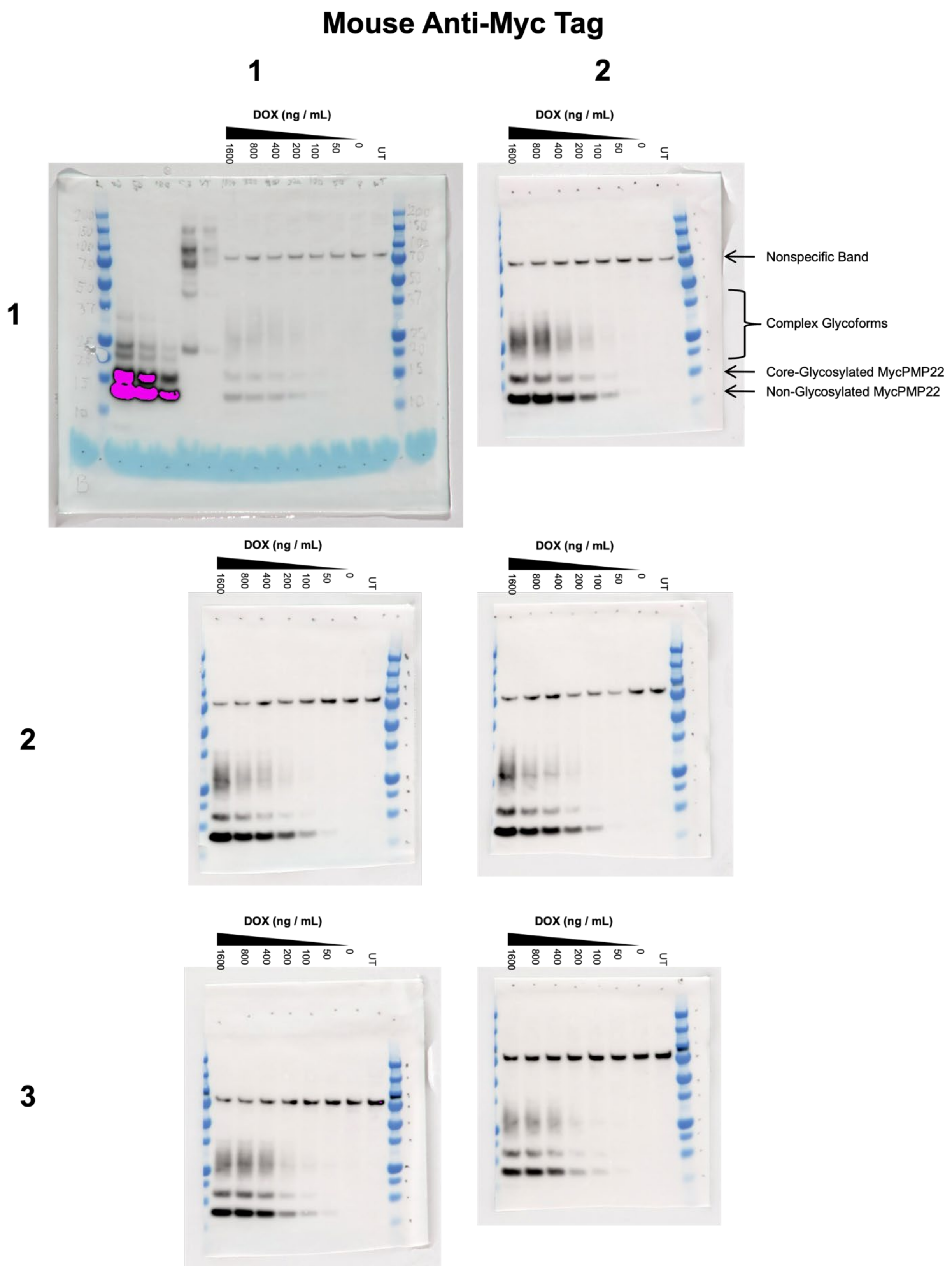

Mouse Anti-Myc Tag
1
2
DOX (ng / mL)
1600
800
400
200
100
50
0
UT
1
2
3
Nonspecific Band
Complex Glycoforms
Core-Glycosylated MycPMP22
Non-Glycosylated MycPMP22

Rabbit Anti-GAPDH

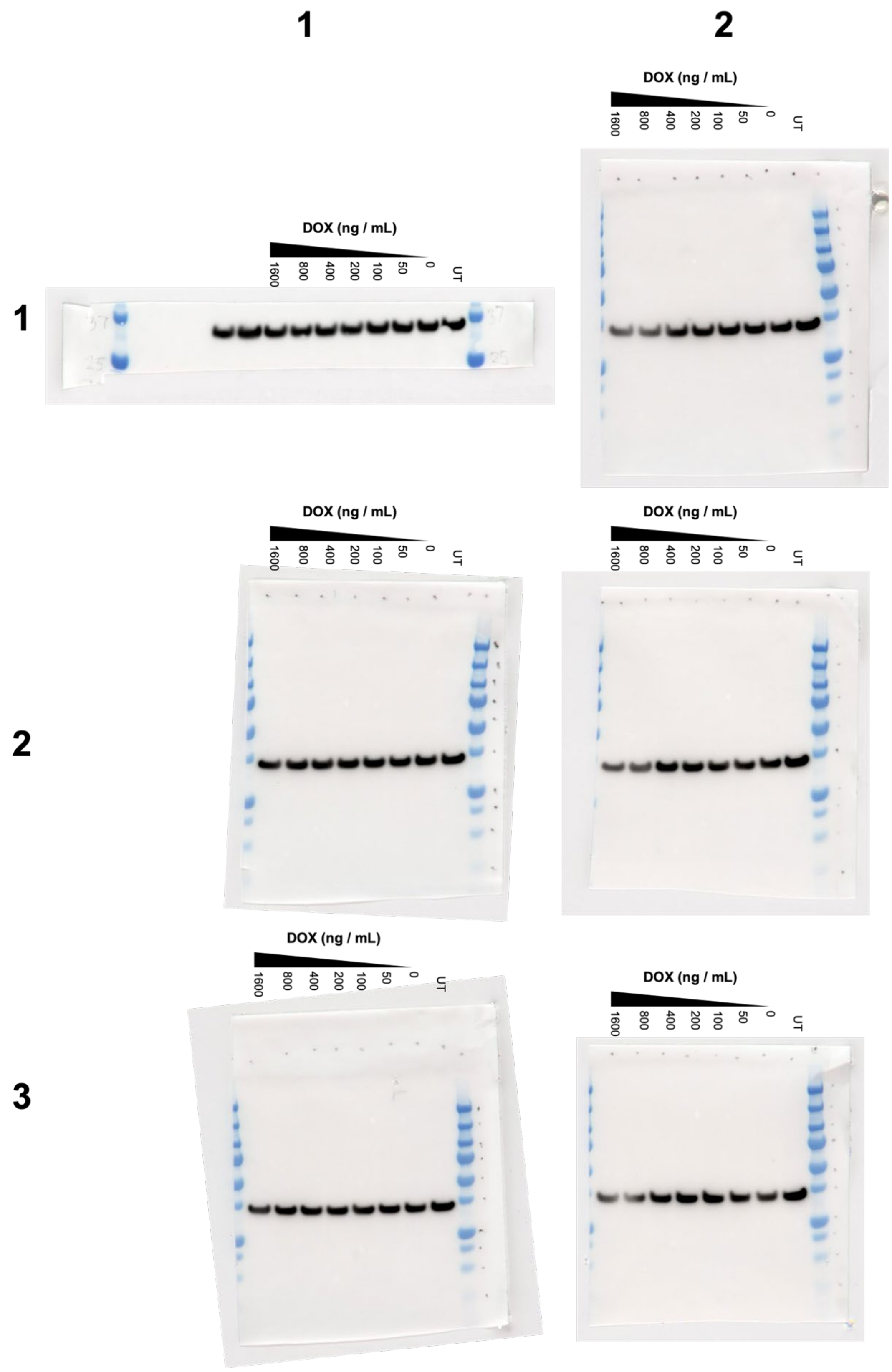

Figure S2. Uncropped membranes and additional GAPDH loading control immunoblot from Figure 1*B*. Biological replicate experiments are shown in rows. Each biological replicate was run on two technical replicate gels (columns). Quantified values for each biological replicate are the average of the two technical replicate blots. Figure 1*C* in the main text depicts biological replicate 2, technical replicate 2 (middle row, right column). 10μg of total lysate protein was loaded per well. The molecular weight marker is Precision Plus Protein™ All Blue Prestained Protein Standard (Bio-Rad, 1610373). Nitrocellulose membranes were first stained with Ponceau S and imaged. After destaining, most blots were then cut prior to immunoblotting. All unlabeled lanes contain samples irrelevant to this work. Membranes were first immunoblotted first using 80 ng/mL anti-myc tag (9B11) mouse monoclonal antibody (Cell Signaling Technology, 2276) and detected with 1:3000-diluted anti-mouse IgG HRP-linked antibody (Cell Signaling Technology, 7076). Membranes were then re-probed with 80 ng/mL anti-GAPDH (14C10) rabbit monoclonal antibody (Cell Signaling Technology, 2118) and detected with 1:3000-diluted anti-rabbit IgG HRP-linked antibody (Cell Signaling Technology, 7074). MycPMP22 glycoforms are labeled as indicated, as is a nonspecific band known to the manufacturer of the anti-myc tag antibody listed above. Note that the observed molecular masses of the mycPMP22 bands in the anti-myc tag immunoblots are lower than expected. For instance, the expected mass of non-glycosylated mycPMP22 is 18 kDa, but we find that it tends to appear between the 10 and 15 kDa markers on the 4-12% gradient polyacrylamide gels we normally use.

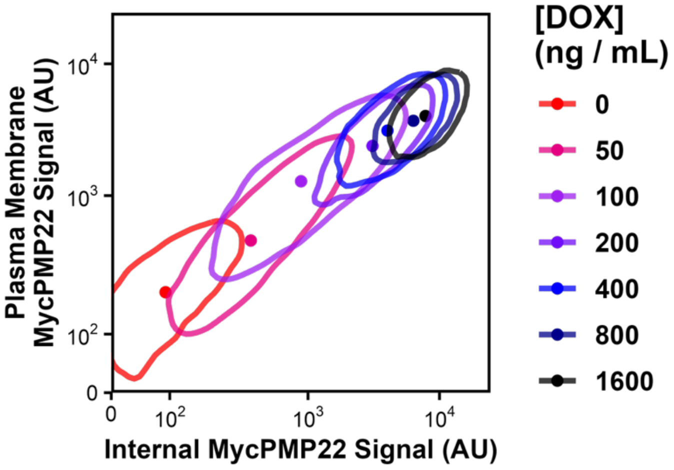

Figure S3. Expanded version of Figure 2 (*Left Panel*) highlighting additional DOX-induced RSC populations. Note that these are background-subtracted values, hence the artifactual negative values observed in the uninduced condition.

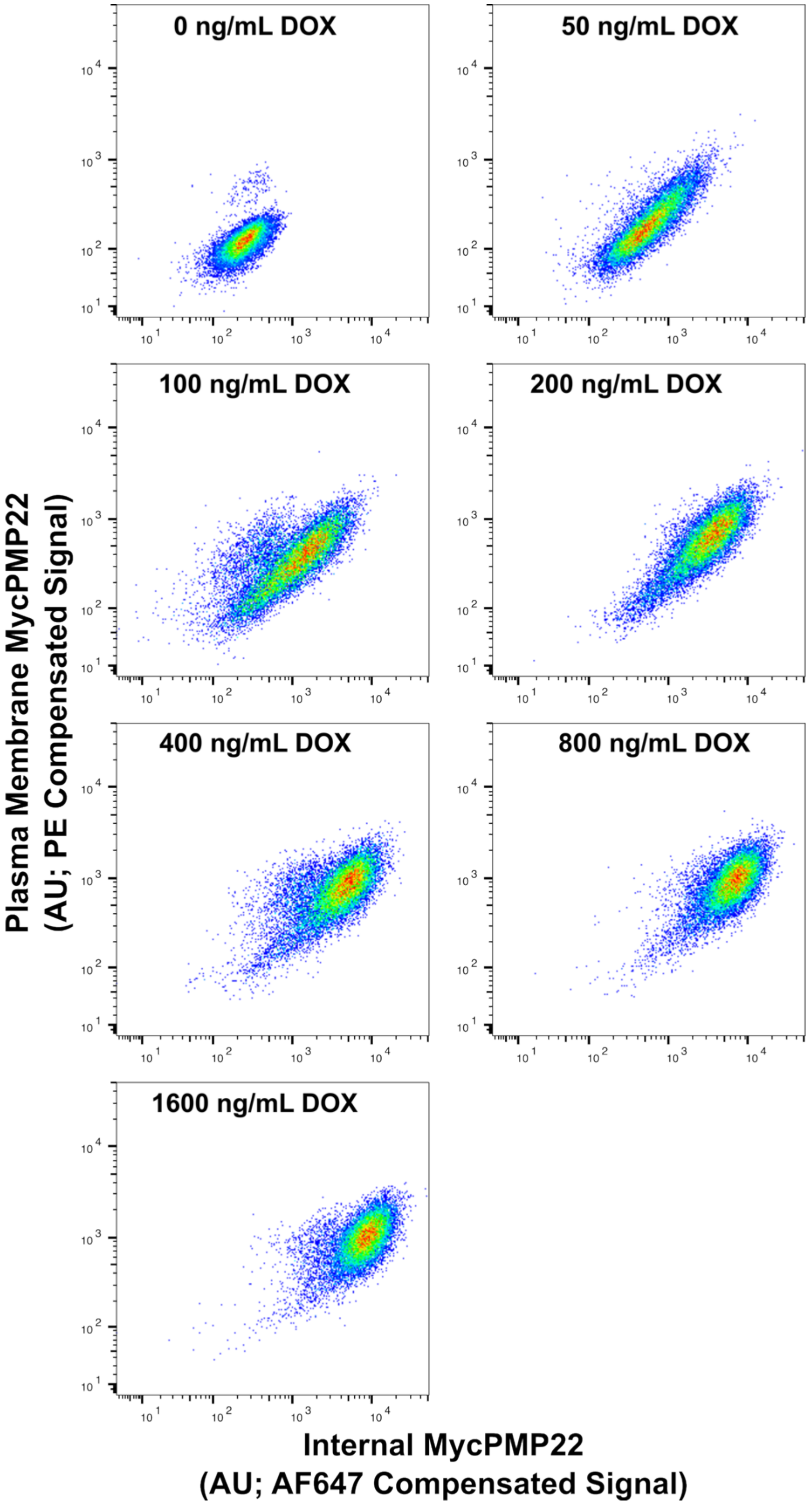

Figure S4. Full scatter plots of raw MycPMP22 immunofluorescence values for each induction condition depicted in Figure 2 (*Left Panel*) and Figure S3. Points are colored according to density (Blue, Low → Red, High). Note that these are raw values obtained by the flow cytometer after fluorophore compensation. No background-subtraction or brightness correction has been applied in these panels.
